# Accelerated interfacial proton transfer for promoting electrocatalytic activity†

**DOI:** 10.1039/d2sc01750d

**Published:** 2022-08-24

**Authors:** Kai-Chao Deng, Zhi-Xuan Lu, Juan-Juan Sun, Jin-Yu Ye, Fan Dong, Hai-Sheng Su, Kang Yang, Matthew M. Sartin, Sen Yan, Jun Cheng, Zhi-You Zhou, Bin Ren

**Affiliations:** State Key Laboratory of Physical Chemistry of Solid Surfaces, Collaborative Innovation Center of Chemistry for Energy Materials (iChEM), College of Chemistry and Chemical Engineering, Xiamen University Xiamen 361005 China bren@xmu.edu.cn sartimm@outlook.com; Fujian Science and Technology Innovation Laboratory for Energy Materials of China China

## Abstract

Interfacial pH is critical to electrocatalytic reactions involving proton-coupled electron transfer (PCET) processes, and maintaining an optimal interfacial pH at the electrochemical interface is required to achieve high activity. However, the interfacial pH varies inevitably during the electrochemical reaction owing to slow proton transfer at the interfacial layer, even in buffer solutions. It is therefore necessary to find an effective and general way to promote proton transfer for regulating the interfacial pH. In this study, we propose that promoting proton transfer at the interfacial layer can be used to regulate the interfacial pH in order to enhance electrocatalytic activity. By adsorbing a bifunctional 4-mercaptopyridine (4MPy) molecule onto the catalyst surface *via* its thiol group, the pyridyl group can be tethered on the electrochemical interface. The pyridyl group acts as both a good proton acceptor and donor for promoting proton transfer at the interfacial layer. Furthermore, the p*K*_a_ of 4MPy can be modulated with the applied potentials to accommodate the large variation of interfacial pH under different current densities. By *in situ* electrochemical surface-enhanced Raman spectroscopy (*in situ* EC-SERS), we quantitatively demonstrate that proton transfer at the interfacial layer of the Pt catalyst coated with 4MPy (Pt@4MPy) remains ideally thermoneutral during the H^+^ releasing electrocatalytic oxidation reaction of formic acid (FAOR) at high current densities. Thus, the interfacial pH is controlled effectively. In this way, the FAOR apparent current measured from Pt@4MPy is twice that measured from a pristine Pt catalyst. This work establishes a general strategy for regulating interfacial pH to enhance the electrocatalytic activities.

## Abstract 2

Adsorbing 4MPy on Pt surface promotes proton transfer at the interfacial layer, maintaining an optimal interfacial pH and promotes electrocatalytic reactions involving proton-coupled electron transfer (PCET) processes.

## Graphical abstract

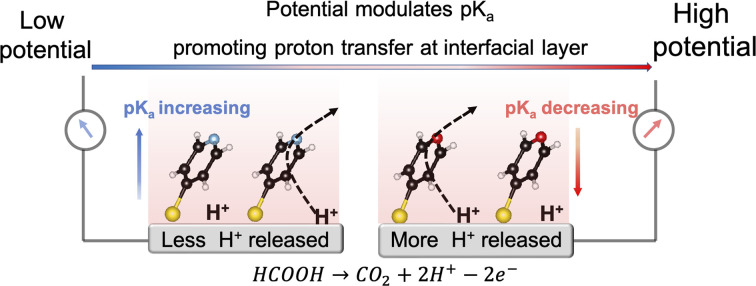

## Introduction

Electrocatalytic reactions play a key role in the interconversion of electrical and chemical energy. The most representative reactions include the O_2_ reduction reaction (ORR),^1–4^ H_2_ oxidation reaction (HOR),^5^ H_2_ evolution reaction (HER),^6,7^ CO_2_ reduction reactions (CO_2_RR),^8–13^ and formic acid oxidation reaction (FAOR).^14–16^ Some steps of these reactions involve the simultaneous transfer of an equal number of electrons and protons, which is called proton-coupled electron transfer (PCET) processes.^17^ The proton transfer implies that the local pH near the electrode influences its reactivity. Qualitatively, a reaction that generates protons as products will perform better at a high pH, whereas one that consumes them will perform better at a low pH. Therefore, an optimal local pH (*i.e.*, proton accepting and donating activity)^18–20^ is needed to achieve the highest conversion efficiency. However, electrocatalytic reactions generating (or consuming) protons at the electrode surface cause the local pH to deviate from the optimal pH for activity.^1,5,8,9,12,21,22^

Controlling the pH at the electrode surface is uniquely challenging and standard buffer solutions have proven insufficient for this purpose.^1,5,8^ For example, during the HOR in phosphate buffer saline (PBS) solution on Pt, Ryu *et al.* measured the pH within the double-layer region by using a non-faradaic probe reaction.^5^ They found that the local pH at the electrode surface was 3.2 units lower than the bulk pH value of 6.8, even at a modest current density (4.6 mA cm^−2^). They proposed that the greatest variation of local pH occurs within the layer with a molecular length scale to the catalyst surface (shortened as interfacial layer in the following text), although the pH gradients are expected to extend into the reaction diffusion layers over micrometer length scales.^5,6,12^ This is because the electrical polarization of the electrode surface in electrochemical reaction processes creates a unique interfacial pH environment at the molecular length scale. Meanwhile, heterogeneous electrocatalytic reactions, especially for inner-sphere reactions, require the binding of reactants to the electrode surface, and consequently, it is the interfacial pH within the reactant molecular length scale that governs the reaction activity. Therefore, it is necessary to regulate the pH effectively in this interfacial layer rather than that in the bulk electrolyte solution to optimize the electrocatalytic activity.

The interfacial pH is dictated by a balance between the proton transport and production (or consumption) rates, and the latter is represented by current density at the electrode surface.^8^ Although the proton transfer by hopping is expected to be fast in electrolyte solution, it involves the rearrangement of many hydrogen bonds and can be strongly affected by nanoconfinement. For example, Bakker *et al.* measured the proton transfer rate in a nanoconfined water droplet of <4 nm and observed that the proton transfer rate is ∼10 times lower than that in bulk solution.^23^ According to Fick's 1st law, the lower mobility of H^+^ results in a higher deviation in H^+^ concentration at the interfacial layer during the reaction. Therefore, the balance of interfacial pH can easily be lost at modest current densities, even when a rotating disk electrode (RDE) is used to accelerate mass transport, leading to loss of control of the interfacial pH. Promoting interfacial proton transfer may be a general way to regulate interfacial pH. Although a few unusual methods were applied to regulate interfacial pH,^12^ such as electrochemical promotion of catalysis, general and effective strategies for regulating the pH environment at the interfacial layer are absent.

**Scheme 1:**
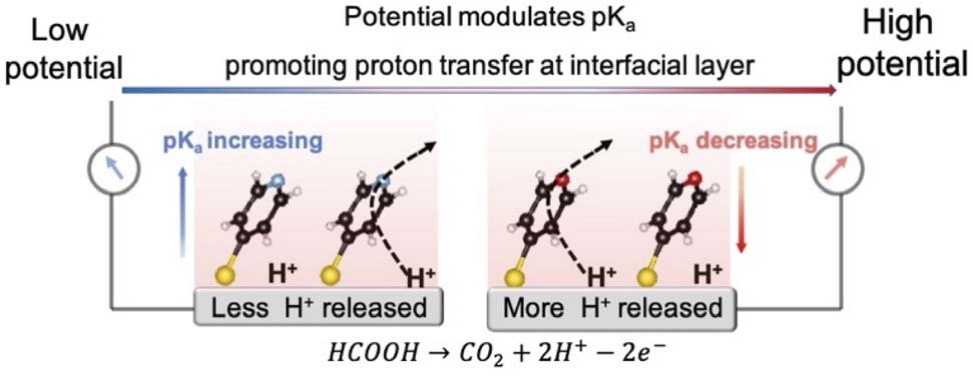
The model for promoting proton transfer by 4MPy at the interfacial layer.

In this contribution, we propose a strategy to promote proton transfer at the interfacial layer to effectively regulate the interfacial pH, thereby enhancing electrocatalytic activity. We accomplish this by adsorbing 4-mercaptopyridine (4MPy) on the catalyst surface as shown in Scheme 1. 4MPy consists of a thiol group, which enables it to immerse in the electrochemical double layer by tethering it near the electrode surface, and a pyridyl group, which can promote proton transfer at the interfacial layer because it is both a good proton acceptor and donor. To demonstrate the effectiveness of this strategy, we use the FAOR on Pt as a model system. The FAOR is a classic inner-sphere reaction involving H^+^ generation at the electrochemical interface and its activity is strongly affected by interfacial pH. By *in situ* EC-SERS, we demonstrate that the ability of 4MPy to promote interfacial proton transfer can be enhanced at more positive potentials (also high current densities) as a result of lowered surface p*K*_a_ of 4MPy at more positive potentials,^24–26^ and thus the interfacial pH environment is regulated. Benefitting from the ability of 4MPy to regulate the interfacial pH, the 4MPy-modified Pt (Pt@4MPy) exhibits a 2-fold greater apparent current for the FAOR than a pristine Pt catalyst, even though only *ca.* 25% of the surface Pt atoms are chemically accessible.

## Results and discussion

### Electrochemical characteristics of the FAOR on pristine Pt and Pt@4MPy

**Fig. 1:**
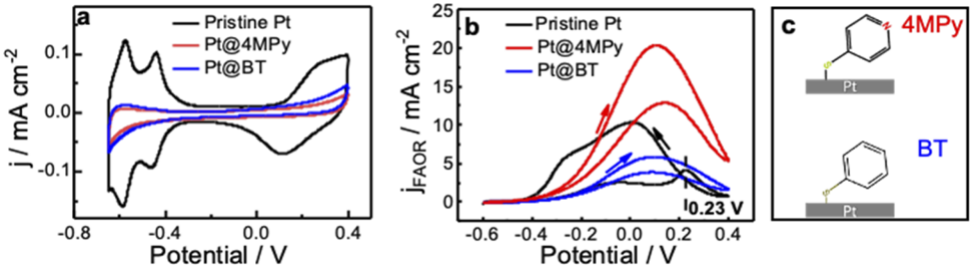
Cyclic voltammograms at 0.1 V s^−1^ on pristine Pt (black curve), Pt@4MPy (red curve) and Pt@BT (blue curve) (a) in 0.5 M H_2_SO_4_ and (b) in 0.5 M H_2_SO_4_ + 0.5 M HCOOH. The scheme of 4MPy and BT on Pt is described in (c). The current density was calculated using the geometric area of the Pt electrode to reflect the effect of 4MPy.

We first demonstrate the electrochemical characteristics of Pt@4MPy with cyclic voltammetry. Fig. 1a shows cyclic voltammograms (CV) obtained at pristine Pt and Pt@4MPy electrodes in 0.5 M H_2_SO_4_. All potentials are referenced to a mercurous sulfate electrode (MSE) instead of a saturated calomel electrode to avoid the influence of Cl^−^. The currents in the hydrogen adsorption/desorption region (<−0.3 V) and Pt oxidation/reduction region (>0 V) are significantly suppressed on Pt@4MPy (red line) compared with that on pristine Pt (black line). By comparing the charges in the hydrogen adsorption/desorption region obtained on Pt and Pt@4MPy, the coverage of 4MPy on the electrode can be estimated.

The voltammetric behavior and FAOR performance of a pristine Pt electrode and the Pt@4MPy electrode in 0.5 M H_2_SO_4_ + 0.5 M HCOOH are compared in Fig. 1b. CV performed on a pristine Pt electrode (black line) shows the characteristic features of a surface poisoned by CO.^27–29^ The sharp peak at 0.23 V corresponds to the oxidation of adsorbed CO. After the removal of CO, a clean Pt surface is exposed for formic acid oxidation, and the reaction current is observed during the negative scan. In contrast to that on the pristine Pt surface, CV performed on Pt@4MPy shows very little hysteresis at the peak potential, and it does not exhibit the CO oxidation peak near 0.23 V. The suppression of CO is further supported by the surface-enhanced Raman spectra (SERS). No signal from adsorbed CO can be detected on Pt@4MPy (see ESI 2.1 for spectra and ESI 2.2 for the preparation of the SERS substrate†). The voltammetric and SERS results indicate that on Pt@4MPy, the formation of adsorbed CO was suppressed, and the HCOOH direct oxidation dominates the FAOR.^30,31^ We recorded the CV curves at different coverages of 4MPy on Pt (ESI 2.3†). The optimal FAOR performance was found at *ca.* 0.75 monolayer coverage of 4MPy on Pt, showing nearly 2-fold apparent current (corresponding to *ca.* 8–10-fold current density, detailed estimation of the electrochemically active surface area (ECSA) can be seen in ESI 2.4†) compared to that measured at pristine Pt. This enhancement is surprising given that, at 0.75 monolayer coverage of 4MPy, only about 25% of the surface Pt atoms are chemically accessible. The improved catalytic activity of Pt@4MPy over pristine Pt may be a result of the site blocking effect, *i.e.*, adsorbed 4MPy blocks the sites necessary for the dissociative formation of CO. Although molecular site blockers have been reported to be able to suppress the CO pathway and enhance the FAOR on Pt, usually the modified surface could only provide 1/2 apparent current (or *ca.* 2-fold aerial current density from the ECSA) than that measured at pristine Pt.^32,33^ Apparently, 4MPy functions more than a site blocking molecule.

### Function of the pyridine group

In order to demonstrate whether the site blocking molecules can fully account for the improved activity of the catalyst, we performed the FAOR at a Pt surface with adsorbed benzenethiol (Pt@BT). BT has nearly the same adsorption configuration and optimal surface coverage as 4MPy,^34^ so it should exhibit a similar site-blocking effect on formic acid reactions to 4MPy. Like Pt@4MPy, the CV of Pt@BT in 0.5 M H_2_SO_4_ + 0.5 M HCOOH shows very little hysteresis (seen in the Fig. 1b blue curve), and CO species were not detected (seen in ESI 2.1†), indicating that the ability of Pt@BT to block the formation of CO is similar to that of Pt@4MPy. However, the current measured at Pt@BT is only 1/4 of that measured at Pt@4MPy (or 1/2 of that measured on pristine Pt) as shown in the Fig. 1b black curve. On considering that the BT molecule has a phenyl group and 4MPy has a pyridyl group, the result may indicate the special function of the pyridyl group on the enhanced FAOR activity at Pt@4MPy.

There are two possibilities to account for the catalytic effect of the pyridyl group of 4MPy on the FAOR. First, the pyridyl group may directly interact with formic acid/formate, helping it to form an active intermediate for the FAOR for understanding the role of pyridine (Py) on Au.^35,36^ However, in our experiment of the FAOR on Pt in the solution containing Py (seen in Fig. S2.5c†), its current measured is lower than that measured on Pt@4MPy. If Pt@4MPy follows the same reason, the FAOR may occur *via* a chemical process followed by an electrochemical process (CE). Second, the CV profiles obtained at different scan rates (from 0.1 to 100 V s^−1^ as shown in Fig. S2.5a†) are almost unchanged, suggesting no such chemical process. Furthermore, EC-SERS results show similar spectra on 4MPy of Pt@4MPy in a solution with and without HCOOH (seen in Fig. S2.5b†), indicating that the interaction between 4MPy and HCOOH can be neglected. These results exclude out the possibility that the pyridyl group interacts with HCOOH to promote the FAOR on Pt@4MPy. Another possibility is that the pyridyl group may promote the activity of the FAOR by accepting H^+^ (FAOR generated), which we discuss in detail in the next section.

### Surface buffering effect of 4MPy

On considering the proposed reaction sequence for the direct oxidation pathway in acidic solution,^14,37^

1 HCOOH ⇌ HCOO^−^ + H^+^

2
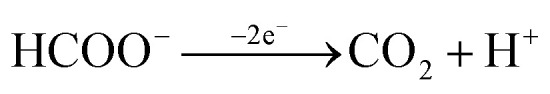
2e^−^/2H^+^ processes are coupled with the oxidation of formic acid, which is confirmed by the shift of the peak potential, *E*_p_, with the variation of pH by −59 mV per pH_bulk_.^14^ Previous studies also show that the activity depends on the solution pH with maximal activity at pH ≈ 4, giving a typical volcano-shape dependence. At higher pH, the adsorbed anions or surface oxides suppress the direct oxidation of formic acid.^14,37,38^ If we are able to introduce a pH buffer species at the interfacial layer, we may be able to tune the activity. As the pyridyl group of 4MPy exists both in protonated and deprotonated forms at different pH, the switch between these two forms makes 4MPy a unique pH buffer agent at the interfacial layer.

**Fig. 2:**
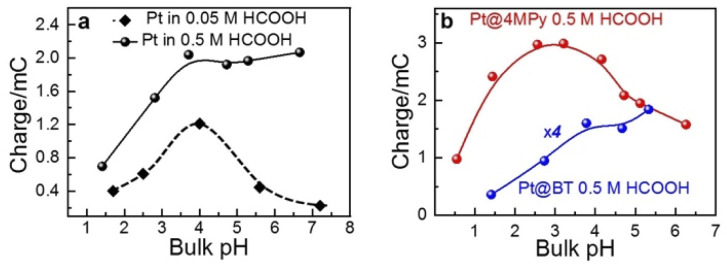
The dependence of the activity on the pH value (a) in 0.05 M (black diamond), 0.5 M (black ball) HCOOH on Pt and (b) 0.5 M HCOOH on Pt@4MPy (red ball) and Pt@BT (blue ball). The activity of the FAOR is characterized using the total charge passing during every CV cycle. The pH was controlled using 0.2 M PBS.

For this purpose, we examined the FAOR activity on Pt with different surface modification, *i.e.*, pristine, 4MPy, and BT, in solutions of different bulk pH. The bulk pH is controlled using 0.2 M PBS. The activity of the FAOR is characterized using the total charge passing during every CV cycle. For a pristine Pt electrode in 0.05 M HCOOH, *E*_p_ shifts by −57 ± 2 mV per pH_bulk_ unit (Table 1), which is in reasonable agreement with the expected peak shift of −59 mV per pH_bulk_. The result indicates that the interfacial pH is similar to bulk. The pH dependence of the activity is shown by the black diamonds in Fig. 2a, which exhibits a volcano-shaped dependence on pH, in which the highest activity occurs at pH ≈ 4, in agreement with previous results for the same system.^14,15,39^ With increasing pH for pH < 4, more of HCOOH deprotonates to form the reactant, HCOO^−^, leading to an increase in activity. The decrease in activity with pH for pH > 4 has been attributed to the site blocking effect by deprotonated phosphate and Pt surface oxidation.^14,37–39^ If the same experiment is performed using 0.5 M HCOOH, the pH-dependent shift in *E*_p_ becomes −42 ± 2 mV per pH_bulk_ unit (Table 1), indicating that the pH at the electrode surface is significantly lower than that in the bulk, due to more H^+^ released during the FAOR at a higher concentration of HCOOH. Moreover, a plot of the FAOR activity *versus* pH shows a plateau at pH > 4, as shown by the black balls in Fig. 2a. Thus, the deviation of the pH-dependent *E*_p_ from −59 mV per pH_bulk_ and the deviation of the activity *vs.* pH plot from a volcano shape indicate that the interfacial pH is out of control, despite the use of the phosphate buffer.

**Table 1:** The dependence of *E*_p_ on bulk pH (details of calculation in ESI 2.6)

Electrode	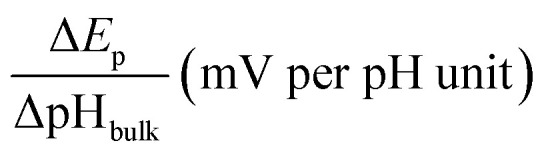
Pt with 0.5 M HCOOH	−42 ± 2
Pt@BT with 0.5 M HCOOH	−45 ± 2
Pt with 0.05 M HCOOH	−57 ± 2
Pt@4MPy with 0.5 M HCOOH	−61 ± 5

When the working electrode was changed to Pt@BT, the *E*_p_ shift becomes −45 ± 2 mV per pH_bulk_ (Table 1), and the FAOR activity *vs.* pH curve, shown in blue balls in Fig. 2b, shows a plateau, indicating that BT does not help regulate the pH at the electrode surface. Interestingly, when Pt@4MPy was used as the working electrode, the *E*_p_ shift is about −61 ± 5 mV per pH_bulk_ (Table 1), showing again the volcano-shaped dependence of activity on pH (red balls in Fig. 2b). The fact that the use of Pt@4MPy makes the pH-dependence of FAOR activity in 0.5 M HCOOH resemble that measured at a pristine Pt electrode in 0.05 M HCOOH demonstrates the effectiveness of 4MPy as a surface buffer.

**Fig. 3:**
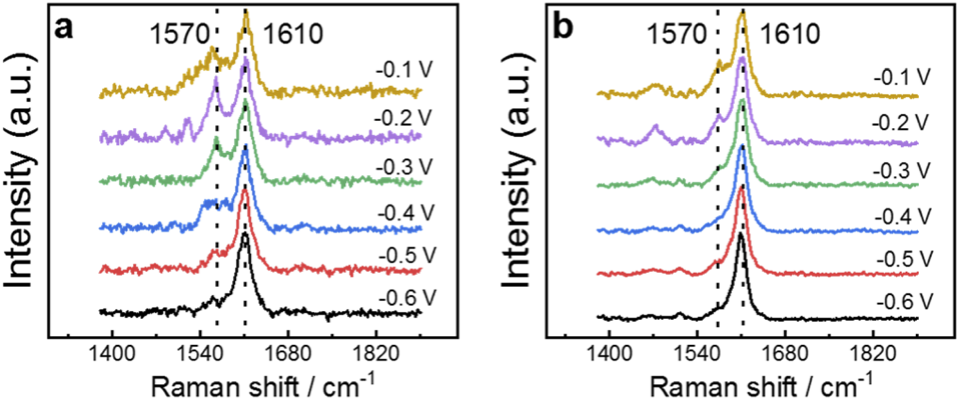
EC-SERS spectra collected at Au@Pt@4MPy while changing the potential from −0.6 to −0.1 V in (a) 0.5 M H_2_SO_4_ and (b) 0.5 M H_2_SO_4_ + 0.5 M HCOOH. The spectra were normalized by the intensity of the 1610 cm^−1^ peak.

The ability of 4MPy to participate in regulating interfacial pH during the FAOR can be observed directly by performing EC-SERS measurements on Au@Pt@4MPy particles during the FAOR. The EC-SERS spectra obtained at several potentials, normalized by the intensity of the 1610 cm^−1^ peak, are presented in Fig. 3a and b. The SERS spectra of 4MPy exhibit peaks at 1570 cm^−1^ and 1610 cm^−1^, which originate from the 8b_2_ ring stretching vibration of the pyridyl of 4MPy in the deprotonated and protonated states, respectively.^40–42^ These bands can be used to indicate the protonation state of 4MPy. In a solution free of HCOOH, a strong signal at 1610 cm^−1^ is observed when the electrode potential is −0.6 V (Fig. 3a), indicating that the protonated form is predominant. As the potential is shifted towards −0.1 V, the peak at 1570 cm^−1^ significantly grows, indicating that 4MPy deprotonates to form the 4MPyH^+^/4MPy buffer pair. Meanwhile, in a solution containing HCOOH, when the FAOR occurs, it releases a large number of protons. The spectral feature at 1610 cm^−1^ that indicates protonation is insensitive to the potential in the range −0.6 V to −0.3 V, as shown by the spectra in Fig. 3b. When the potential reaches −0.2 V, the 1570 cm^−1^ band becomes visible and is weaker than in 0.5 M H_2_SO_4_ solution. The results indicate that 4MPy continuously accepts protons from the reaction. Interestingly, when the potential reaches −0.2 V, the 1570 cm^−1^ band becomes visible, indicating that at sufficiently positive potential, 4MPy can also deprotonate to form the 4MPyH^+^/4MPy buffer pair during FAOR processes. It is due to the potential-dependent decrease in p*K*_a_ as a result of the electronic effect.^25,26,43^ This effect is central to understanding how 4MPy regulates interfacial pH and will be discussed in the next section.

### Interfacial proton transfer model

Clearly, 4MPy adsorbed on Pt can regulate the interfacial pH in FAOR processes, due to the buffering effect of 4MPyH^+^/4MPy. However, when FAOR occurs at the Pt surface, it produces a large number of protons, leading to a maximal pH fluctuation within molecular length scales near the electrode surface. As the number of produced protons is 2–3 orders of magnitude higher than that of 4MPy molecules on the Pt surface (details of calculation in ESI 2.7†), the surface buffering effect of 4MPy alone does not have the capacity to continually bind with H^+^. An alternative mechanism for regulating interfacial pH is to promote proton transfer away from the interfacial area as occurring.^17^

**Fig. 4:**
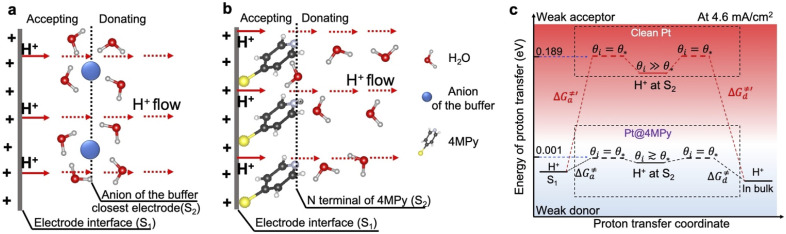
Interfacial models showing proton transfer at the interfacial layer of (a) Pt and (b) Pt@4MPy. The S_1_ plane represents the electrode surface. The S_2_ plane represents the molecular layer closest to the electrode surface. An efflux of protons (marked by the red arrow) is generated at S_1_ and passes through the S_2_ plane. (c) The value of the free energy diagram for proton transfer at the interfacial layer at high current densities (for example 4.6 mA cm^−2^). The black lines (reaction on Pt@4MPy) and red lines (reaction on clean Pt) show the thermodynamic pathways for proton transfer at the interfacial layer. The Δ*G*^≠^_a_ and Δ*G*^≠^_d_ represent the energy barrier for accepting and donating protons at *θ*_i_ = θ_*_.

To demonstrate how 4MPy promotes proton transfer, we develop a simplified interfacial model to describe the proton transfer at the interfacial layer of clean Pt and Pt@4MPy. The schematics of the interfacial proton transfer mechanisms for each catalyst are depicted in Fig. 4a and b, respectively. For simplicity, we only show the additional H^+^ (FAOR generated) and the molecular layer closest to the electrode surface. The S_1_ plane represents the electrode interface, where the FAOR occurs in an inner-sphere way to produce H^+^ (not yet hydrated). The S_2_ plane represents the anion of the buffer solution closest to the clean Pt electrode, or the N terminal of 4MPy on the Pt@4MPy electrode. During a steady-state FAOR process, H^+^ generated at the S_1_ plane will be accepted and then donated by molecules at the S_2_ plane, and finally transferred along the hydrogen-bonded network driven by a pH gradient and electric field gradient.

The Stokes–Einstein law indicates that the mobility of H^+^ is affected by its interaction with surrounding molecules. Therefore, the capacity of accepting and donating proton acceptors at the S_2_ plane plays a crucial role in proton transfer processes at the interface. For example, in unbuffered solutions, the individual H_2_O molecules and H_2_O clusters are weak proton acceptors at the S_2_ plane, because of p*K*_a_ (H_3_O^+^) = −1.74. This leads to low mobility of H^+^.^23^ Conversely, a weak donor (strong acceptor) at the S_2_ plane would hinder continuous transfer of protons. As a result, the species should be both good proton acceptors and donors at the S_2_ plane to promote proton transfer.

A change of Gibbs free energy can quantitatively describe the requirements of H^+^ accepted and donated at the S_2_ plane as follows (detailed derivation seen in ESI 2.8†):3
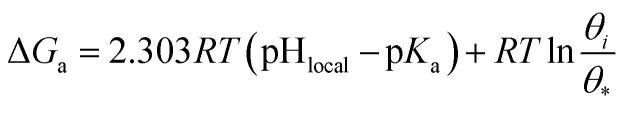
4
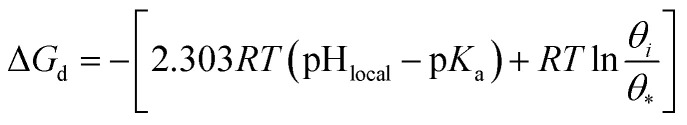
where p*K*_a_ is the acid dissociation constant of molecules at the S_2_ plane. Δ*G*_a_ and Δ*G*_d_ correspond to a change of free energy in accepting and donating protons, respectively. As Δ*G*_a_ equals −Δ*G*_d_, in further analysis we only focus on the analysis of Δ*G*_a_. The *θ*_i_ is the numbers of proton-occupied sites and θ_*_ is the numbers of free sites at the S_2_ plane. A measurement of the inherent capacity of molecules to accept/donate protons should be under the same conditions. Here, we assumed *θ*_i_ = θ_*_, corresponding to a hypothetical state shown in Fig. 4c, and the change of Gibbs free energy for accepting protons can be simplified as:

5 Δ*G*^≠^_a_ = 2.303*RT*(pH_local_ − p*K*_a_)

indicating a highly effective thermodynamic pathway of proton transfer with Δ*G*^≠^_a_ ∼ 0 at pH ≈ p*K*_a_ and *vice versa* (shown in Fig. 4c) as similar trend to literature.^20,44^ This thermodynamic expression is consistent with the general theory of acid–base buffering, which states that buffering capacity is at its maximum at pH ≈ p*K*_a_ and half of buffer molecules are protonated. The model can therefore explain how the buffering effect in our system results from the promotion of proton transfer.

**Fig. 5:**
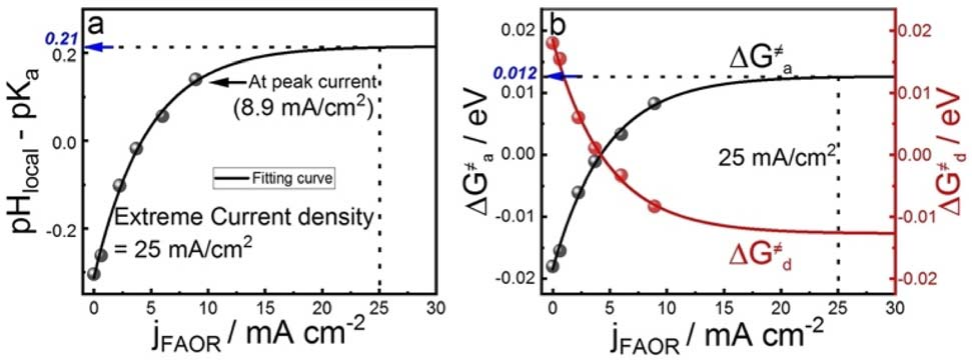
The dependence of the (a) pH_local_ − p*K*_a_ of 4MPy and (b) Δ*G*^≠^_a_ Δ*G*^≠^_d_ of proton transfer on the current densities.

To understand the effect of 4MPy on improving the reaction activity, we plot pH_local_ − p*K*_a_ and Δ*G*^≠^_a_ under different reaction currents in Fig. 5a. pH_local_ − p*K*_a_ was directly obtained from experimentally obtained SERS spectra by taking the ratio of the intensities of 1570 and 1610 cm^−1^ bands of 4MPy during the voltammetric process (detailed CV-SERS method and calculation are provided in ESI 1.6 and 2.9†). The current densities are directly obtained from the voltammogram shown in ESI 2.10.† As shown in Fig. 5a, the value of pH_local_ − p*K*_a_ remains small between −0.30 and 0.14 as the current density increases from 0 to 8.9 mA cm^−2^, which is much smaller than the large variation of the interfacial pH (ca. 3.2 units change) in the PBS buffer solutions.^5^ Thus, according to eqn (5), it is easily understood that the proton transfer is more effective on Pt@4MPy than on clean Pt in PBS solutions because the former experiences a lower energy barrier (shown in Fig. 4c black dots) than the latter (shown in Fig. 4c red dots). Furthermore, the fitting result shows an exponential dependence of *j* with pH_local_ − p*K*_a_. Considering *j* ∝ exp(Δ*EαF*/*RT*) ^14,37^and Δ*E* ∝ (pH_local_ − p*K*_a_),^24,26^ we can easily obtain *j* ∝ exp[(pH_local_ − p*K*_a_)*αF*/*RT*]. From the fitting results, pH_local_ − p*K*_a_ remains to be 0.21 even at a current density of 25 mA cm^−2^ for practical concentration of formic acid in a fuel cell (the current density of the cyclic voltammogram shown in ESI 2.11†). Under these conditions, the Δ*G*^≠^_a_ is only about 0.012 eV (Fig. 5b), which only leads to 11% increase of the fraction of protonated 4MPy compared with that under *θ*_i_ = θ_*_ conditions. Therefore, the excellent performance of 4MPy can be attributed to its strong ability to promote the proton transfer and regulate pH at the interfacial layer.

**Fig. 6:**
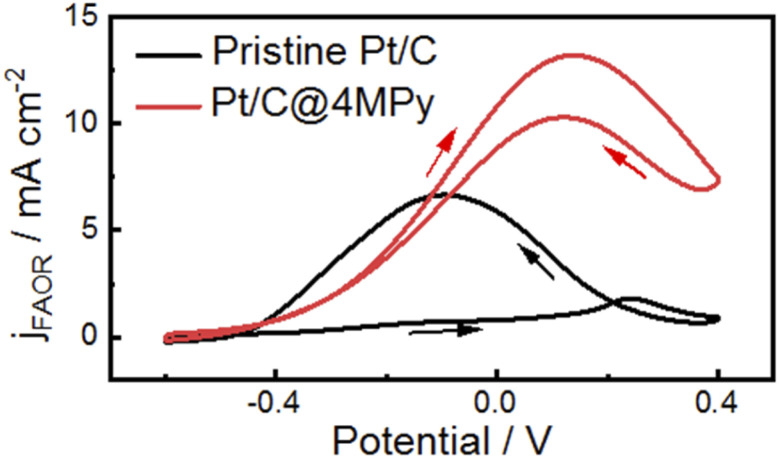
Cyclic voltammograms at 0.1 V s^−1^ on pristine Pt/C (black curve) and Pt/C@4MPy (red curve) in 0.5 M H_2_SO_4_ + 0.5 M HCOOH. The current density was calculated using the geometric area of the electrode.

The approach of improving proton transfer at the interfacial layer can be applied to commercial catalysts to enhance their electrocatalytic activity. We demonstrate the applicability by modifying a commercial Pt/C electrode with 4MPy for the FAOR (preparation method given in ESI 1.3†). Fig. 6 shows cyclic voltammograms recorded in 0.5 M H_2_SO_4_ + 0.5 M HCOOH using the 4MPy-modified Pt/C catalyst (termed Pt/C@4MPy, red curve). A control experiment was performed on the Pt/C catalyst (black curve). From the voltammograms, the peak current measured at Pt/C@4MPy is about 2 times higher than that measured at Pt/C, similar to that on planar Pt@4MPy. We furthermore applied Pt@4MPy for methanol oxidation reaction (MOR). We observed much higher enhancement in catalytic activity than that predicted by the site-blocking effect, as a result of the promoted proton transfer in the presence of 4MPy. This result convincingly demonstrates an effective approach to regulate interfacial pH by promoting proton transfer at the interfacial layer, thereby enhancing the electrocatalytic activity.

## Conclusions

In summary, we proposed a general strategy for promoting proton transfer at the interfacial layer in order to regulate the interfacial pH and enhance the electrocatalytic activity. We promoted the electrocatalytic activity of the FAOR on Pt by surface modification with 4MPy (Pt@4MPy). In addition to the site blocking effect to prevent the formation of poisoning CO species, the higher activity of the Pt@4MPy catalyst is mainly due to the lowered p*K*_a_ of 4MPy at more positive potentials, which leads to a better buffering effect of the 4MPyH^+^/4MPy pair and more effective regulation of the interfacial pH. Such a promotion effect mainly results from the promotion of proton transfer at the interfacial layer by 4MPy, which is different from the general acid–base buffering system. We further described the proton transfer processes in a thermodynamic way and quantitatively obtained the energy barrier of proton transfer on Pt@4MPy at different current densities of the FAOR on Pt@4MPy. The energy barrier is only about 0.012 eV and therefore the proton transfer process remains thermoneutral, even at a very high current density of 25 mA cm^−2^. These results well explain the effectiveness of 4MPy as a surface buffer over a wide range of current densities. Benefitting from this unique property, Pt@4MPy exhibited 2-fold apparent current (or about 8–10-fold current density by the estimation of the ECSA) for the FAOR than a pristine Pt catalyst, even though only *ca.* 25% of the surface Pt atoms are chemically accessible. We further applied this modification strategy to a commercial Pt/C catalyst, which also showed 2-fold improved FAOR activity. The present study demonstrates a general strategy for promoting proton transfer at the interfacial layer and thereby effectively enhancing the electrocatalytic activity, which can be applied to other heterogeneous catalytic reactions, such as MOR, CO_2_RR, and ORR.

## Data availability

The data that support the findings of this study are available from the corresponding authors only upon reasonable request.

## Author contributions

The manuscript was written through contributions of all authors. All authors have given approval to the final version of the manuscript.

## Conflicts of interest

The authors declare no competing financial interest.

## Supplementary Material

SC-013-D2SC01750D-s001
