# Supplementary material for: Accelerated interfacial proton transfer for promoting electrocatalytic activity
Source: Chem Sci. 2022 Aug 24;13(36):10884–90. doi: 10.1039/d2sc01750d (PMC9491081; doi:10.1039/d2sc01750d)
Supplement: SC-013-D2SC01750D-s001 [file SC-013-D2SC01750D-s001.pdf]

Electronic Supplementary Material (ESI) for Chemical Science.
This journal is © The Royal Society of Chemistry 2022

Supporting Information

Accelerated interfacial proton transfer for promoting the electrocatalytic activity

Kai-Chao Deng, [a] Zhi-Xuan Lu, [a] Juan-Juan Sun, [a] Jin-Yu Ye, [a,b] Fan Dong, [a] Hai-Sheng Su, [a] Kang Yang, [a] Matthew M. Sartin*, [a] Sen Yan, [a] Jun Cheng, [a,b] Zhi-You Zhou, [a,b] Bin Ren* [a,b]

[a]State Key Laboratory of Physical Chemistry of Solid Surfaces, Collaborative Innovation Center of Chemistry for Energy Materials (iChEM), College of Chemistry and Chemical Engineering, Xiamen University, Xiamen 361005, China;

[b]Fujian Science and Technology Innovation Laboratory for Energy Materials of China

Author to whom correspondence should be addressed: bren@xmu.edu.cn; sartimm@outlook.com

Table of Content

1. Experimental Procedures....4

1.1 Chemicals and materials....4

1.2 Preparation of Pt@4MPy and Pt@BT electrodes....5

1.3 Preparation of Pt/C@4MPy electrodes....5

1.4 Preparation of 140 nm Au@Pt NPs....5

1.5 Electrochemical measurements....5

1.6 In situ-electrochemical SERS measurements....5

1.7 Calculating the coverage of 4MPy on Pt....6

2. Supporting data....6

2.1 Raman spectra on clean Pt, Pt@4MPy and Pt@BT in 0.5 M H_2SO_4 + 0.5 M HCOOH....6

2.2 SEM image of 140 nm Au@Pt....6

2.3 The dependence of Pt@4MPy activity on 4MPy coverage....6

2.4 Activity of FAOR by electrochemically active surface area (ECSA) calculated....7

2.5 Excluding out the possibility of the pyridyl group interacting with HCOOH....7

2.6 Dependence of E_p on the bulk pH....7

2.7 Estimating the number of 4-MPy molecules on surface compared with the protons released from FAOR....8

2.8 The derivation of change of Gibbs free energy in proton accepting and donating processes..8

2.9 Calculating pH_{local} - pK_a of 4MPy....8

2.10 A voltammogram of FAOR on Pt@4MPy during in-situ EC-SERS....9

2.11 A voltammogram of Pt@4MPy in 0.5 M H_2SO_4 + 5 M HCOOH....9

2.12 Cyclic voltammograms of MOR on Pt and Pt@4MPy....9

2.13 EC-SERS spectra collected at Au@Pt@4MPy in solutions with 0.05 and 5 M HCOOH.....10

2.14 Performance of different materials on FAOR....10

References....11

1. Experimental Procedures

1.1 Chemicals and materials

4-mercaptopyridine (4MPy, 95%), benzenethiol (BT, 90%) and ascorbic acid (AA, 99%) were purchased from Sigma-Aldrich (Shanghai, China). AR grade Formic acid, H_2SO_4, $Na_2HPO_4 \cdot 12H_2O$, $Na_2HPO_4 \cdot 2H_2O$, Chloroauric acid (≧ 47.8%, w%/Au), and Chloroplatinic (≧ 37.0% w%/Pt) were purchased from Sinopharm Chemical Reagent Co., Ltd. Nafion (5% w/w in water) and 20 wt% Pt/C were purchased from Alfa Aesar. All electrolyte solutions were prepared using Millipore water (18.2 MΩ).

1.2 Preparation of Pt@4MPy and Pt@BT electrodes

The pristine polycrystalline Pt electrodes were cleaned in the 0.5 M H_2SO_4 solution by repetitive potential scans between surface oxidation and the hydrogen adsorption regions until a stable CV was obtained. Then, the clean Pt electrodes were immersed in ethanolic solutions of 4MPy (or BT) to allow the formation of self-assembled monolayers (SAM) on the surfaces. The coverage of 4MPy on Pt was controlled by varying the adsorption concentration of 4MPy. The samples were rinsed with ethanol and dried under N_2. Then the samples were directly used for experiments.

1.3 Preparation of Pt/C@4MPy electrodes

Commercial 20 wt% Pt/C catalyst (2 mg) were dispersed into a solution consisting of 0.5 mL ultra-pure (18 MΩ) water, 0.5 mL ethanol, and 0.05 mL 5% Nafion. The ink (2 μL) was dropcast on a glassy carbon electrode (diameter: 2 mm) and then dried in vacuum. Then, the Pt/C electrode was immersed in a solution of 2.5-5 μM 4MPy in water for 10-12 hours to allow the formation of a self-assembled monolayer (SAM) of 4MPy on the Pt catalyst surface.

1.4 Preparation of 140 nm Au@Pt NPs

140 nm Au nanoparticles were synthesized according to a previously reported method.[1] 40 mL of the 140 nm Au colloid was heated to 80 °C in a water bath. Then, 1.87 mL solutions of 1 mM H_2PtCl_6 and 5 mM ascorbic acid were simultaneously, slowly injected into the 140 nm Au colloid over the course of 10 min. After complete injection, the reaction was allowed to continue for 30 min at 80 °C yielding approximately 1 nm Pt shell on the gold nanoparticles.[2]

1.5 Electrochemical measurements

Electrochemical measurements were performed using a standard three-electrode glass cell and a potentiostat (CHI 660e). The Pt@4MPy samples (prepared by the methods described in 1.2) and the clean Pt electrodes were used as the working electrodes in the respective experiments. Because we are studying the anodic processes, the dissolution of platinum on the cathodic Pt counter electrode can be avoided and therefore a Pt wire can be used as the counter electrode. A mercurous sulfate electrode (MSE, Hg/Hg_2SO_4, saturated K_2SO_4) was used as the reference electrode and it can be found from Figure S1.1 that the MSE is very stable compared with NHE.

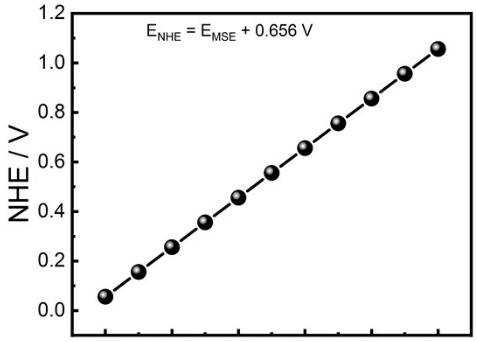

Figure S1.1 Reference calibration plot of MSE vs NHE.

1.6 In situ-electrochemical SERS measurements

In situ-electrochemical SERS measurements were performed on a WITec Alpha 300 R confocal Raman system. A water immersion objective with a numerical aperture of 1.0 and magnification of 60 was used for laser illumination and signal collection. The 632.8 nm excitation was generated using the output from a He-Ne laser, and the power of the laser on the sample was about 0.2 mW. The spectroelectrochemical cell was equipped with a Pt wire as the counter electrode, and MSE was used as the reference electrode.[3] During the synchronized CV-SERS, EMCCD generated a transistor−transistor logic (TTL) signal right before its exposure, which was sent to the potentiostat to initiate the potential sweep. Thereby, the current and spectral data can be exactly synchronized, and every SERS spectrum in the CV-SERS spectra and every current point in CV can be well correlated.[4]

1.7 Calculating the coverage of 4MPy on Pt

The coverage of 4MPy (θ_{4MPy}) on Pt can be calculated using the following equation:

$$\theta_{4MPy} = \frac{S_{Pt(ECA)} - S_{Pt@4MPy\,(ECA)}}{S_{Pt(ECA)}}$$

where $S_{Pt(ECA)}$ and $S_{Pt@4MPy(ECA)}$ are the electrochemically accessible areas of the pristine and 4MPy-coated Pt electrodes, respectively, obtained from the charge in the hydrogen adsorption and desorption regions.

2. Supporting data

2.1 Raman spectra on clean Pt, Pt@4MPy and Pt@BT in 0.5 M H_2SO_4 + 0.5 M HCOOH

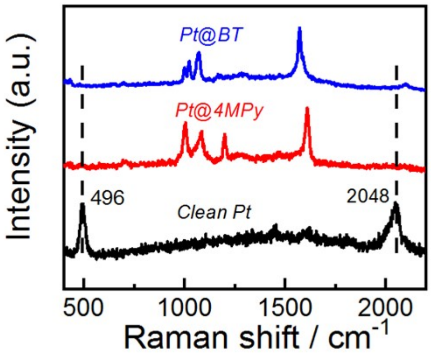

Figure S2.1 Raman spectra of pristine Pt (black spectrum), Pt@4MPy (red spectrum) and Pt@BT (blue spectrum) in 0.5 M H_2SO_4 + 0.5 M HCOOH at open circuit potential (OCP).

2.2 SEM image of 140 nm Au@Pt

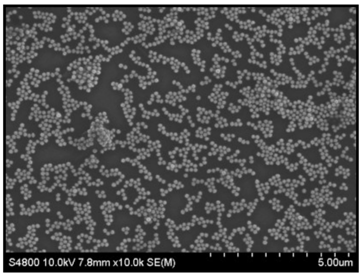

Figure S2.2 SEM image of 140 nm Au@Pt NPs used as SERS substrate.

2.3 The dependence of Pt@4MPy activity on 4MPy coverage

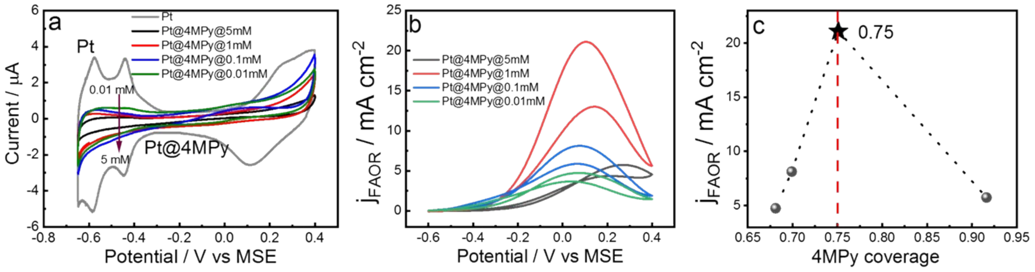

Figure S2.3 Cyclic voltammograms of pristine Pt (black line) and Pt@4MPy electrode with different coverages of 4MPy in (a) 0.5 M H_2SO_4 and (b) 0.5 M HCOOH + 0.5 M H_2SO_4. Scan rate: 0.1 V/s. (c) The dependence of the current density on 4MPy coverage. The different coverages of 4MPy were controlled by immersing in different 4MPy concentrations. The asterisk represents the optimal coverage of about 0.75. The method for calculating 4MPy coverage is described in section 1.7. The current density was calculated using the geometric area of the Pt electrode.

2.4 Activity of FAOR by electrochemically active surface area (ECSA) calculated

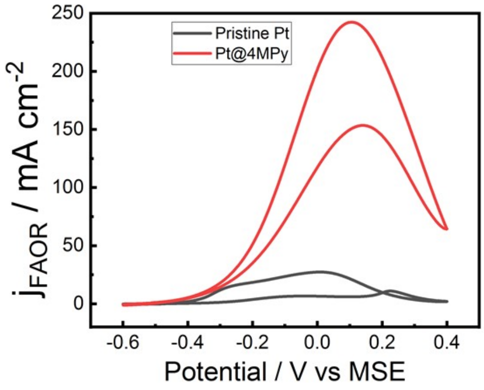

Figure S2.4 Cyclic voltammograms at 0.1 V/s on pristine Pt (black curve), Pt@4MPy (red curve) in 0.5 M H_2SO_4 + 0.5 M HCOOH. The current density was calculated using electrochemically active surface area (ECSA). The data is from Figure 1b, and the ECSA is calculated by the charge in the hydrogen adsorption and desorption regions in Figure 1a.

2.5 Excluding out the possibility of the pyridyl group interacting with HCOOH

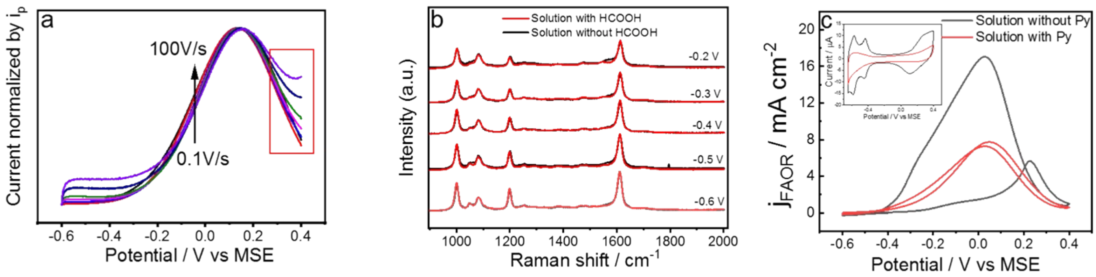

Figure S2.5 (a) Voltammograms of Pt@4MPy with increasing scan rate from 0.1 V to 100 V/s. The CVs are normalized by peak current. The rising at the end of curves (marked by the red rectangle) is result from diffusion control under the high scan rates. (b) EC-SERS spectra collected at Au@Pt@4MPy while changing the potential from -0.6 to -0.2 V in 0.5 M H_2SO_4 (black spectra) and 0.5 M H_2SO_4 + 0.5 M HCOOH (red spectra). (c) Cyclic voltammograms at 0.1 V/s on Pt in solution of 0.5 M H_2SO_4 + 0.5 M

HCOOH without pyridine (black) and with 20 mM pyridine (red). Inset: Cyclic voltammograms at 0.1 V/s on Pt in solution of 0.5 M without pyridine (black) and with pyridine (red). The current density was calculated using the geometric area of the Pt electrode to reflect the effect of Py.

2.6 Dependence of E_p on the bulk pH

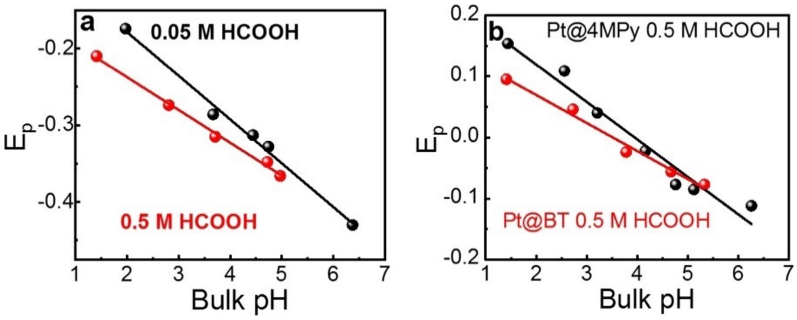

Figure S2.6 The dependence of peak potential (E_p) on the bulk pH value (a) in 0.05 M (black), 0.5 M (red) HCOOH on clean Pt and (b) 0.5 M HCOOH on Pt@4MPy (black) and Pt@BT (red).

2.7 Estimating the number of 4-MPy molecules on surface compared with the protons released from FAOR

Assuming a value of 1.8×10^{15} molecules/cm^2 for the surface concentration of chemisorbed 4MPy at most.[5] Therefore, the number of molecules were about 0.56×10^{14} molecules on a Pt electrode (diameter 2 mm). According to Faradic law, the number of 0.61×10^{16} protons was released even for the lowest activity in pH = 0.61 (seen in Figure 2b). The number of protons by FAOR released is about 2-3 orders of magnitude higher at least than the number of 4MPy molecules on Pt surface.

2.8 The derivation of change of Gibbs free energy in proton accepting and donating processes

$$H^+ + A \overset{1/K_a}{\Leftrightarrow} HA^+ \overset{K_a}{\Leftrightarrow} H^+ + A \quad (1)$$

The above equation describes proton accepting and donating processes by the buffer molecules. Where K_a is the acid dissociation constant of molecules. The change of Gibbs free energy can be described as follows:

$$\Delta G_a = \Delta G_a^\theta + RT\ln\frac{[HA^+]}{[H^+]_{local}[A]} \quad (2)$$

where ΔG_a and ΔG_a^θ are the change of Gibbs free energy and standard Gibbs free energy when accepting protons. With

$$\Delta G_a^\theta = RT\ln K_a \quad (3)$$

We obtain:

$$\Delta G_a = RT\ln K_a + \mathrm{RT}\ln\frac{[HA^+]}{[H^+]_{local}[A]} = 2.303RT(pH_{local} - pK_a) + RT\ln\frac{\theta_i}{\theta_*} \quad (4)$$

Where the θ_i and θ_* are numbers of proton occupied sites and free sites. The derivation for proton donating processes is the same as proton accepting processes, and is described as follows:

$$\Delta G_{a,d} = \pm \left[2.303RT(pH_{local} - pK_a) + RT\ln \frac{\theta_i}{\theta_*} \right] \quad (5)$$

2.9 Calculating $pH_{local} - pK_a$ of 4MPy

The value of $pH_{local} - pK_a$ can be determined using the Henderson-Hasselbach equation:

$$pH_{local} - pK_a = \log \frac{[4MPy]}{[4MPyH^+]} \quad (6)$$

$$[4MPy]/[4MPyH^+] = ([I_{1570}]/[I_{1610}])\alpha \quad (7)$$

To obtain $pH_{local} - pK_a$, we calculated the quantity [4MPy]/[4MPyH$^+$] as a function of applied potential by using the ratio of the intensities of 1570 and 1610 cm^{-1} bands obtained from the EC-SERS experiments to represent their respective concentrations by equations (6) and (7). α is a dimensionless number of conversion factor between concentration and Raman intensity. α *was* measured by comparing the intensity ratio, $I_{4MPy}/I_{4MpyH}{}^+$, measured by NMR (Figure S2.5 a) with that measured by Raman (Figure S2.5 b) in a same acidified 4MPy solution partially. The α was 3.2.

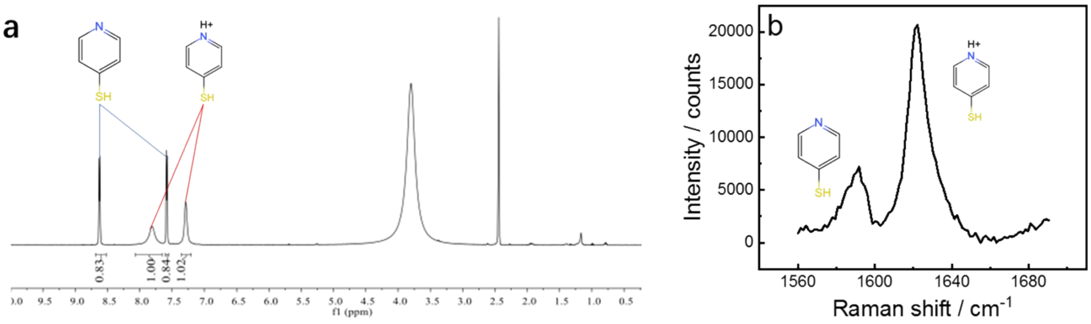

Figure S2.7 (a) An NMR and (b) Raman spectrum of partially acidified 4MPy solution.

2.10 A voltammogram of FAOR on Pt@4MPy during in-situ EC-SERS

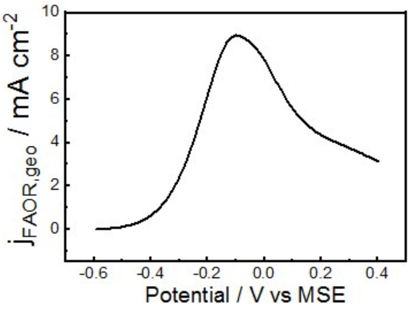

Figure S2.8 A voltammogram of Au@Pt@4MPy in 0.5 M H_2SO_4 + 0.5 M HCOOH. The current density was calculated using the geometric area of the Pt electrode. The current density was calculated using the geometric area of the Pt electrode.

2.11 A voltammogram of Pt@4MPy in 0.5 M H_2SO_4 + 5 M HCOOH

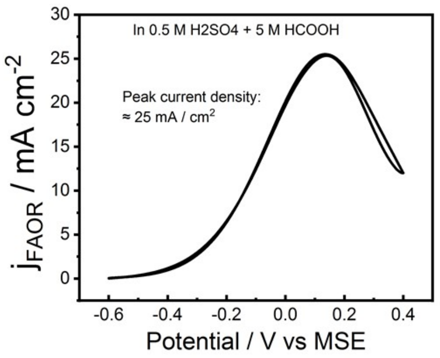

Figure S2.9 A voltammogram of Pt@4MPy in 0.5 M H_2SO_4 + 5 M HCOOH. The current density was calculated using the geometric area of the Pt electrode

2.12 Cyclic voltammograms of MOR on Pt and Pt@4MPy

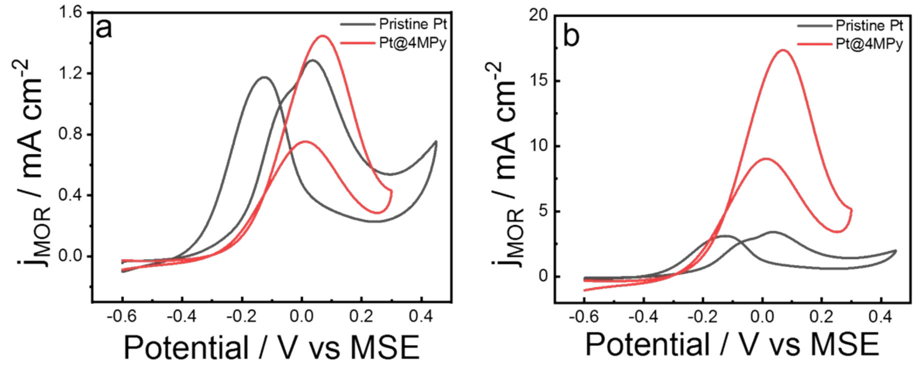

Figure S2.10 Cyclic voltammograms at 0.1 V/s on pristine Pt (black curve) and Pt@4MPy (red curve) in solution containing 0.5 M methanol at pH ≈ 4.4. The current densities were calculated using (a) geometric area and (b) electrochemically active surface area (ECSA) of Pt.

2.13 EC-SERS spectra collected at Au@Pt@4MPy in solutions with 0.05 and 5 M HCOOH

The EC-SERS spectra results show similar features for Pt@4MPy in solutions with different HCOOH concentration at -0.6 V. However, with the positive shift of the potential, we found the appearance of 1570 cm^{-1}, indicating that deprotonation appears at different potential in the solutions with containing different HCOOH concentration. In the solution with 0.05 M HCOOH, the 1570 cm^{-1} peak could be clearly observed at -0.5 V. In the solution with 0.5 M HCOOH, the 1570 cm-1 band becomes visible at -0.2 V (see Figure 3b). In the solution with 5 M HCOOH, the 1570 cm^{-1} band is hardly observed even at potentials more positive than -0.1 V. These results indicate that the 4MPy remains protonated because a large number of protons release at interface during the FAOR in HCOOH solution with high concentration. It agrees with the conclusion in the Figure 3.

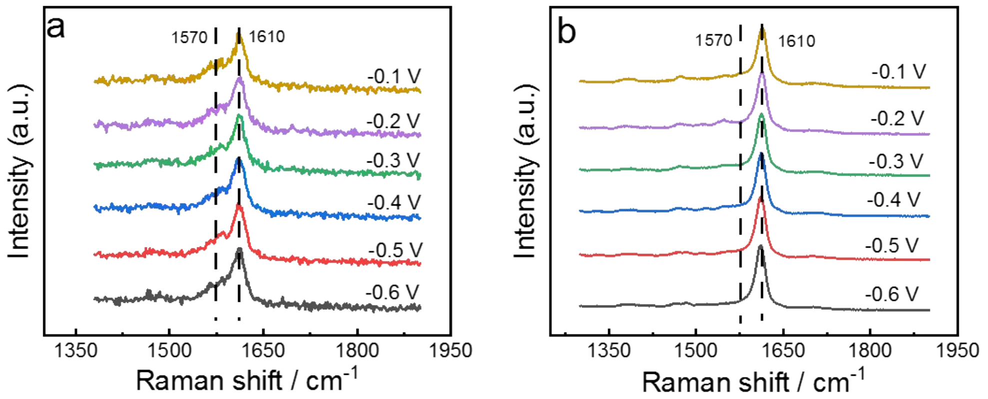

Figure S2.11 EC-SERS spectra collected at Au@Pt@4MPy while changing the potential from -0.6 to -0.1 V in solution with (a) 0.05 HCOOH and (b) 5 M HCOOH.

2.14 Performance of different materials on FAOR

Table S1. A comparison of FAOR activity of our work with that reported in references.[6-9] The current densities were normalized by GSA (geometric surface area) and ECSA (electrochemically active surface area)

Catalyst	Electrolyte + formic acid	Scan rate V/s	Peak Current (GSA) mA/cm^2	Peak Current (ECSA) mA/cm^2	Reference
Pt@4MPy	0.5 M H_2SO_4 + 0.5 M HCOOH	0.1	**20**		our work
Pt@4MPy	0.5 M H_2SO_4 + 0.5 M HCOOH	0.1		**240**	our work
Pt（111）@Bi	0.5 M $HClO_4$ + 0.05 M HCOOH	0.05		2.5	Ref.6
Pt (111)@CN^-	0.1 M $HClO_4$ + 0.2 M HCOOH	0.5		60	Ref.7
PtBi	0.1 M $HClO_4$+0.125 M HCOOH	0.01	3.8		Ref.8
Pt@FeTSPs	0.1 M $HClO_4$ + 0.1 M HCOOH	0.1		12	Ref.9
PtPb	0.1 M $HClO_4$+0.25 M HCOOH	0.01	8.2		Ref.8

References

1. Ziegler, C.; Eychmüller, A., Seeded Growth Synthesis of Uniform Gold Nanoparticles with Diameters of 15−300 nm. The Journal of Physical Chemistry C 2011, **115**, 4502-4506.

2. Tian, Z.-Q.; Ren, B.; Li, J.-F.; Yang, Z.-L., Expanding generality of surface-enhanced Raman spectroscopy with borrowing SERS activity strategy. Chemical Communications 2007, 3514-3534.

3. De-Yin Wu, J.-F. L., Bin Ren and Zhong-Qun Tian, Electrochemical surface-enhanced Raman spectroscopy of nanostructures. Chemical Society Reviews 2008, **37**, 1025-41.

4. Zong, C.; Chen, C.-J.; Zhang, M.; Wu, D.-Y.; Ren, B., Transient Electrochemical Surface-Enhanced Raman Spectroscopy: A Millisecond Time-Resolved Study of an Electrochemical Redox Process. Journal of the American Chemical Society 2015, **137**, 11768-11774.

5. Aggarwal, R. L.; Farrar, L. W.; Saikin, S. K., Increase of SERS Signal upon Heating or Exposure to a High-Intensity Laser Field: Benzenethiol on an AgFON Substrate. The Journal of Physical Chemistry C 2012, **116**, 16656-16659.

6. Perales-Rondón, J. V.; Ferre-Vilaplana, A.; Feliu, J. M.; Herrero, E., Oxidation Mechanism of Formic Acid on the Bismuth Adatom-Modified Pt (111) Surface. Journal of the American Chemical Society 2014, **136**, 13110-13113.

7. Cuesta, A.; Escudero, M.; Lanova, B.; Baltruschat, H., Cyclic Voltammetry, FTIRS, and DEMS Study of the Electrooxidation of Carbon Monoxide, Formic Acid, and Methanol on Cyanide-Modified Pt(111) Electrodes. Langmuir 2009, **25**, 6500-6507.

8. Casado-Rivera, E.; Volpe, D. J.; Alden, L.; Lind, C.; Downie, C.; Vázquez-Alvarez, T.; Angelo, A. C. D.; DiSalvo, F. J.; Abruña, H. D., Electrocatalytic Activity of Ordered Intermetallic Phases for Fuel Cell Applications. Journal of the American Chemical Society 2004, **126**, 4043-4049.

9. Zhang, Z.; Zhou, X.; Liu, C.; Xing, W., The mechanism of formic acid electrooxidation on iron tetrasulfophthalocyanine-modified platinum electrode. Electrochemistry Communications 2008, **10**, 131-135.
